# Differential Interspecific Adaptation to Abiotic Stress by *Plantago* Species

**DOI:** 10.3389/fpls.2020.573039

**Published:** 2020-11-05

**Authors:** António Teixeira, Peter E. Toorop, Pietro P. M. Iannetta

**Affiliations:** ^1^Department of Comparative Plant and Fungal Biology, Royal Botanic Gardens, Kew, Richmond, United Kingdom; ^2^Department of Earth and Environmental Sciences, University of Pavia, Pavia, Italy; ^3^Ecological Sciences, The James Hutton Institute, Dundee, United Kingdom

**Keywords:** *Plantago*, seed germination, seedling establishment, abiotic stress, conservation

## Abstract

The success of seed-based conservation and restoration efforts using native plant species is largely determined by ensuring two key life history transitions are accommodated. These are from “seed to germinated seed” and “germinated seed to established seedling.” In turn, optimization of these life history transitions is determined by a “genetic × environmental” interaction and later largely characterized by localized climatic (abiotic) conditions. It is these environmental stress factors that can act as natural selection agents for specific plant–trait combinations, or phenotypes. In turn, such adaptation may also limit a species range. To test the relationship between these two early plant life history stage transitions, “seed to germinated seed” and “germinated seed to established seedling,” the attributes were characterized for two species of *Plantago* that occupy contrasting environments and since these species have potential for native seed-based habit restoration and conservation. The species were *Plantago coronopus* (L.), localized at lower and drier altitudes, and *Plantago lanceolata* (L.), characterized as occupying higher and wetter altitudinal clines. Seeds were collected from 20 accessions of six natural populations spanning four European countries for both *P. lanceolata* and *P. coronopus*. Seed germination (*G*) and seedling establishment (*S*) data were determined at six temperatures (*T*) and six water potentials (Ψ), and the data obtained were analyzed using a generalized linear model (GLM). The results indicate that *P. coronopus* has adapted physiologically to its high-altitude conditions such that seed germination and seedling establishment may be more readily achieved in this cooler environment where water is less limiting. In contrast, the lower θ_T_ of *P. lanceolata* better facilitates more efficient seed germination and seedling establishment in drier and warmer clines of lower altitude. In addition to establishing a genotypic (species) underpin for seed and seedling trait differences observed, the insights gained may also be exploited to best deploy each species *in situ* for seed-based conservation and restoration efforts.

## Introduction

Land abandonment is one of the major threats to the Mediterranean biodiversity (Myers et al., 2000) and is mainly the result of socioeconomic changes where less productive areas, which are also less accessible due to steep topography and/or poor road conditions, have been particularly badly affected (Loumou and Giourga, 2003; Rühl et al., 2005). In addition, crop monocultures and the intensive use of agrochemicals, which characterize conventional food production systems, leave soils degraded and often bare soils (e.g., olive groves and vineyards) throughout the year, leading to erosive processes and loss of soil nutrients. In semiarid zones where majority of the Mediterranean agro ecosystems are located, restorative agronomic approaches using cover crops (Dazy et al., 2008) would help improve soil function (Dölle and Schmidt, 2009; Moreno-de Las Heras et al., 2009) and help realize a more sustainable system for the long-term (Hobbs and Norton, 1996). One restorative agriculture approach for mainly perennial cropping systems is the reestablishment of wild native or indigenous species using seed-based methodologies. However, the success of deploying wild seed in such restorative efforts demands detailed species- and even ecotype-specific knowledge of seed and seedling traits and their response to local environmental factors (Hernández and Pastor, 2008).

Ambient temperatures and water availability are major factors influencing seed germination and seedling establishment (Finch-Savage and Phelps, 1993; Xu et al., 2014; Cochrane et al., 2015; Dürr et al., 2015; Teixeira et al., 2020). Concerning the former, thermal time models of germination rates in response to temperature may be used to estimate “*cardinal temperatures*,” which define temperature minima or “*base temperature*” (*T*_*b*_), below which seeds will not germinate (Garcia-Huidobro et al., 1982; Gummerson, 1986; Dahal and Bradford, 1994; Hardegree and Van Vactor, 1999). The cardinal temperature approach also identifies the temperature at which germination is optimal (*T*_*o*_) and the ceiling temperature when germination is prevented (*T*_*c*_) (Ellis et al., 1986; Alvarado and Bradford, 2002). The *T*_*b*_ is a critical parameter for predictive modeling of the periods during which seed germination is possible (Trudgill et al., 2005), and seeds produced by a community of any given species can show tight regulation of their *T*_*b*_ (Garcia-Huidobro et al., 1982; Covell et al., 1986; Dahal et al., 1990; Kebreab and Murdoch, 1999). Thus, cardinal temperatures can relate seed germination data to germination and to seedling development to indicate the potential occurrence of mature plants (Alvarado and Bradford, 2002) and fulfillment of ecological niche (that is, the match of a phenotype to specific environmental conditions; *c.f.*, Arnaud, 2015).

Regarding water availability, changes in soil water content affect soil water potential and so may affect water transport to, and germination of, seeds (Guillemin et al., 2013; Teixeira et al., 2020). The base water potential (Ψ_*b*_) is a critical parameter for use in hydrotime models to predict the germination periods of different species (or ecotypes) in a variety of environmental conditions (Bradford, 1990; Welbaum et al., 1990; Guillemin et al., 2013; Dürr et al., 2015).

In a study conducted to estimate *T*_*b*_ and Ψ_*b*_ parameters of 14 species, Guillemin et al. (2013) reported that the number of potential “germination days” varied little among the climatic years tested but substantially between species. That is, species coexistence appears afforded based on differential ecological niches. In terms of ecological restoration and especially restoration activities, which focus upon the use of native seeds from a range of local species, germination traits and predictive models are especially important since the success of restoration, and species coexistence, may depend on seed germination and seedling establishment (Bischoff et al., 2006).

The genus *Plantago* (Plantaginaceae) is thought to be an ancient family (Kuiper and Bos, 1992) and is here reported on two common herbaceous ruderal species that tend toward being perennial types: *Plantago coronopus* with therophyte life form and *Plantago lanceolata* with therophyte or hemicryptophyte life form. The two species occur widely and appear to lack a strong habitat preference, are found in altitude ranges of 1,100 and 2,300 m, and have distributions spanning Eurasia and occidental Europe, respectively (Castroviejo, 1986–2015). The rosette-forming species are pioneer species of disturbed bare ground (Kuiper and Bos, 1992). Naturally occurring and persistent in the soil seed bank, *Plantago* species are used for seed-based landscape restoration (Schröder and Prasse, 2013). Particularly, *P. lanceolata* is frequently used in seed cover crop mixtures (Alvarez-Diaz et al., 2016; González et al., 2017) protecting soil and to control pernicious weed species of farmland, and they are also grazing-tolerant (Lawson et al., 2004). *P. coronopus* is also widely used on well-drained soils, as it is able to establish well despite the potential reduction in available water compared to other soil types (González et al., 2017).

To test the hypothesis that congeneric species may possess distinct traits as adaptations to abiotic stress, i.e., may occupy different ecological niches, we characterized the seed and seedling traits of 10 accessions for each of the two species that were collected from six regions of four countries. The accessions were localized across an array of different altitudes, latitudes, longitudes, and pedoclimatic environments.

## Materials and Methods

### Plant Material

Seeds from two species *P. coronopus* and *P. lanceolata* were collected from 50 to 200 plants per accession in 2015, totaling 20 accessions in six regions covering four countries: Canary islands (Ci, Spain), Andalucía (An, Spain), Minho and Trás-os-Montes (PT, Portugal), Asturias (As, Spain), Sussex (Eg, United Kingdom), and Provence-Alpes-Côte d’Azur (Fr, France). The accessions were sampled at the time of natural seed dispersal (late-summer, Supplementary Table S1). Upon collection, seed of all accessions were cleaned and kept in a controlled environment [15°C, 15% relative humidity (RH)] until germination experiments were performed.

### Climatic Conditions

For each accession, we obtained the climatological conditions by cross-referencing their geographic localities with the WORLDCLIM database (Hijmans et al., 2005) using DIVA-GIS software (Hijmans et al., 2004). With the obtained interpolated climatological data, we calculated the average annual temperature (AT) plus the maximum (T_max_) and minimum (T_min_) temperatures. We also recorded average annual precipitation (AP), the monthly average from November to April [or “winter precipitation” (WP)] and May to October [or “summer precipitation” (SP)], and the sum of the annual precipitation (AP_sum_; Supplementary Table S1). A map of the collecting sites was made using The Environmental Stratification of Europe dataset (QGIS Version 2.10.1; Metzger, 2018) with QGIS software version 2.10 (Open Source Geospatial Foundation Project) (Supplementary Figure S1).

### Seed Mass

The seed mass of each accession was recorded as the mean of 96 seeds selected randomly and weighed individually with a precision balance (UMT2 *d* = 0.1 μg, METTLER TOLEDO, Leicester, United Kingdom).

### Germination and Seedling Development

Laboratory germination (*G*) experiments were performed in temperature-controlled incubators (LMS, Sevenoaks, United Kingdom). Seeds were sown on 9-cm-diameter Petri dishes on two layers of filter paper (Whatman^®^ Grade1, Sigma Aldrich, Gillingham, United Kingdom) and saturated with 3.5 ml of sterile Milli-Q^®^ water (Merck Millipore, Watford, United Kingdom) and placed in closed plastic bags to prevent evaporation. Each replicate comprised a single Petri dish containing 50 seeds, and three replicate experiments were performed independently. Germination temperature regimes ranged from 5 to 30°C at 5°C intervals at a constant temperature and a 12-h light/12-h dark photoperiod. Germination was scored twice daily for 5 weeks, and germinated seeds were distinguished by protruded radicles (>1 mm). Germinated seeds were monitored every 3 days to score the date of cotyledon opening and were considered seedlings when the developing shoot meristem was visible and any abnormality was absent (NS). At the end of the test period, seeds that had failed to germinate were cut open and classified by appearance as viable or dead depending of embryo presence/absence (Teixeira et al., 2020).

Germination percentages were calculated, and statistical analyses were undertaken using only viable seed (i.e., total germinated plus ungerminated living seed).

The capacity of the seed to germinate under simulated conditions of water deficit was assessed using polyethylene glycol (PEG) solutions of varying concentrations as *osmoticum*. These tests were performed in three independent replicate experiments using 3.5 ml of PEG solution ranging from 0 to −1.0 MPa with 0.2-MPa intervals. Seeds were incubated at constant temperature in filter paper-containing Petri dishes (as described above) and at a constant temperature of 15°C. Each solution was prepared in triplicate according to Equation (5) described in Hardegree and Emmerich (1990), Equ. 1 (0.129 [PEG]^2^ T – 14.0 [PEG]^2^ – 0.40 [PEG]). Where “T” is the temperature for germination and “PEG” is MW 8,000 (Sigma Aldrich, Gillingham, United Kingdom). The Petri dishes were placed in closed plastic bags; one dish per treatment per experiment. Each Petri dish containing 50 seeds sown was scored daily, and seeds with protruded radicles >1 mm were considered to have completed germination. In order to keep the osmotic solutions at the designated water potential, the germinated seeds were carefully transferred to germination paper in transparent resealable plastic boxes (17 cm × 12 cm × 5 cm), which was moistened with 15 ml of the same osmotic solution. The boxes were also placed inside sealable plastic bags. The growing seedlings (*S*) were observed every 3 days to score cotyledon opening. As before, and at the end of the test, non-germinated seeds were cut open and classified by appearance as viable or dead depending on embryo presence/absence, germination percentages were calculated, and further statistical analyses (see below) were undertaken only with viable seeds (germinated plus remaining viable).

### Statistical Analysis

Statistical tests were performed using R v3.6.2 (R Core Team, 2016). The medians and quartiles of the boxplots were calculated using the R-Basic functions. “Germination rate” (GR_T_ in response to differing temperatures and GR_W_ for different osmotica) was inferred from the reciprocal of the time to 50% germination (t50), which was estimated *via* a sigmoidal curve fit of the cumulative germination for each dish using the Boltzmann equation (Supplementary Figure S2a). Using the range of constant temperatures of each replicate experiment per accession, they were regressed with a linear model to estimate the “base temperature” at which the germination rate was equal to zero (*T*_*b*_G), and “thermal time” (θ_*T*_G) was estimated with the reciprocal of the (temperature × 1/t50) slope value (Supplementary Figure S2b). The “seedling rate” (SR) was inferred from the reciprocal of the time until 50% of the germinated seeds had open cotyledons (at the constant temperature). The same procedure was used to estimate the base temperature (*T*_*b*_), at which the rate of development to the seedling stage (for temperature and osmoticum, SR_*T*_ and SR_*W*_, respectively) was equal to zero (T_*b*_G and T_*b*_S, respectively). The thermal time for this transition (θ_*T*_S) was also estimated as already described above. To estimate the base water potential (Ψ_*b*_) at which the GR was equal to zero and the “hydrotime” (θ_*H*_; time for transition to seedling stage), a similar procedure to that described above was applied using a range of water potentials (i.e., *osmotica*) to give estimates of θ_*H*_G and θ_*H*_S, respectively (Supplementary Figures S2c,d). To estimate the temperatures and osmotica at which the germination and development to the seedling stage percentages were the highest, data were fitted with quadratic polynomial equations using the Origin software (OriginLab, Northampton, MA, United States). This identified the optimum temperatures for germination (*T*_*o*_G) and for seedlings occurrence (*T*_*o*_S).

The GLM tests for maximum germination and development to the seedling stage were performed using a binomial distribution with logit link function, with species as predictor factor using the maximum likelihood estimation method, Wald Chi-Square statistics, and contrast pairwise species. The GLM tests of the base temperatures, thermal and hydrotime, optimum temperatures, and base water potential were performed using the above method with a normal distribution and the identity link function after normality and homogeneity of the variances checking using the Shapiro–Wilk and Levene tests, respectively. All GLM tests were performed with the SPSS Statistics for Windows software (Version 21.0). Germination rates and normal seedling development rates were tested using the Kruskal–Wallis test, comparing temperature conditions and *osmoticum* using the SPSS Statistics for Windows software (Version 21.0). The base temperatures and thermal time for germination (*T*_*b*_G and θ_*T*_G, respectively) were regressed against the base temperatures and thermal times for normal seedling development (*T*_*b*_S and θ_*T*_S, respectively), the base water potentials and hydrotime for germination (Ψ_*b*_G and θ_*H*_G, respectively) were regressed against the base water potential and hydrotime for normal seedling development (Ψ_*b*_S and θ_*H*_G, respectively), using linear regression with the mean values of the three replicates for each accession and species using the R basic functions. Base temperatures and water potentials were regressed against thermal and hydrotime, and the *T*_*b*_G, *T*_*b*_S, Ψ_*b*_G, and Ψ_*b*_S were regressed against the climate traits T_*min*_, T_*max*_, WP, and SP. The two-way ANOVA tests comparing species’ *T*_*b*_G with *T*_*b*_S and Ψ_*b*_G with Ψ_*b*_S were performed using the SPSS Statistics for Windows software (Version 21.0). The factor analysis of mixed data (FAMD) was performed using the mean values of the three replicates in each physiological trait and the above-described data for geographical and climate conditions as numerical variables with species and region as categorical variables. All numerical variables were centered and scaled to homogenize high discrepancies. The FAMD was performed using the R FactoMineR package (version 2.0). The Spearman’s rank correlation was performed for individual selected species with the means of the three replicates of all treatments. The double correlation matrix of Spearman’s rank and *p*-values was produced with the R package corrplot (version 0.84) using the correlation matrix (adjusted for ties) and the exact probabilities values previously calculated with the GenStat software (VSN International, Hemel Hempstead, United Kingdom).

Graph theory, a mathematical object corresponding to a network that recently gained much attention, was used to analyze ecological attributes such as biological interaction webs, gene–protein interrelations, and flower and pollinator interactions (Proulx et al., 2005). The approach considers a set of units, called nodes or vertex, connected by edges where nodes represent a component of the network and edges indicate a relationship between them (Windram and Denby, 2015). The graph network analysis of traits was performed with the significant Spearman’s rank *p*-values in order to obtain a network graph of physiologic and environmental traits using the significant *rho* traits, where the significant *p* values in the graph correspond to the edges and the traits correspond to the nodes in an adjacency matrix. The *rho p* values of geographical vs. geographical traits and geographical vs. climate traits were excluded from the analysis. The weighted and undirected graph was generated with the R package igraph (version 1.0.1).

## Results

### Temperature Response

**FIGURE 1:**
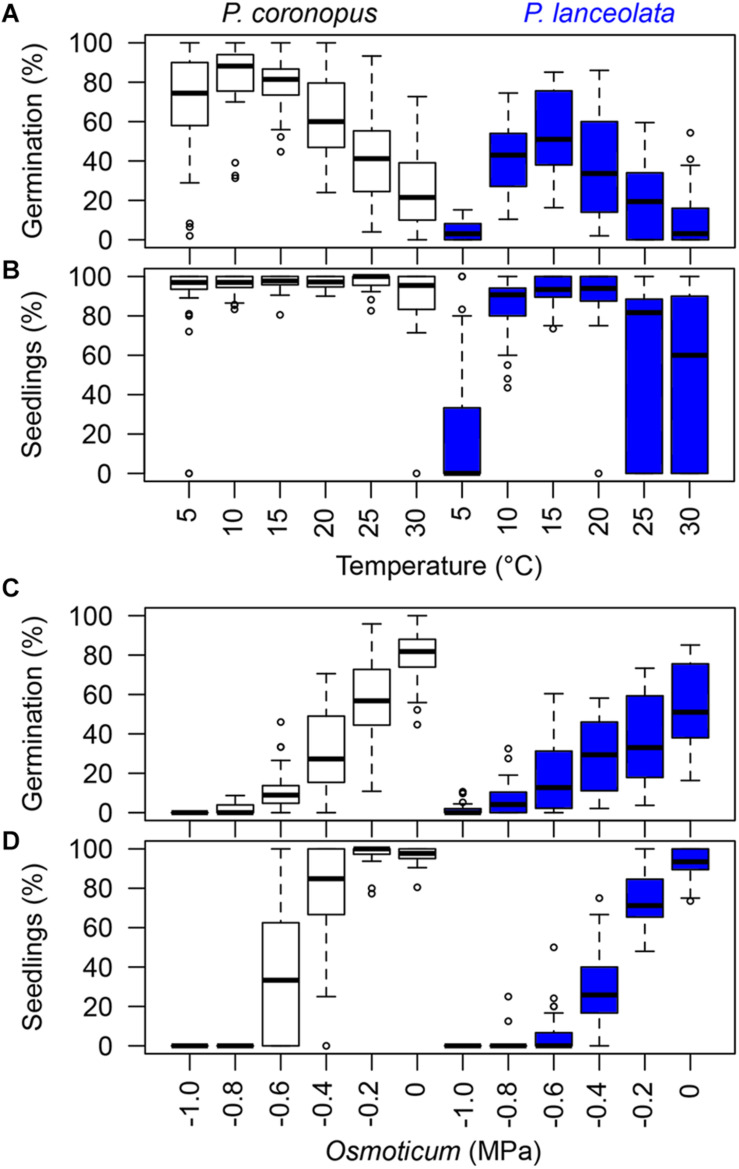
Seed germination percentages calculated from the total viable sowed seeds and normal seedlings as percentage of all germinated seeds, expressing the relative conversion from seed germination to normal seedlings. The boxplots show the minimum, first quartile, median, third quartile, maximum, and the suspected outliers for the germination and seedlings in temperature conditions **(A,B)** and germination and normal seedlings in *osmoticum* **(C,D)**. The boxplot colors represent the different species with 10 accessions each: *P. coronopus* (white); *P. lanceolata* (blue).

Significant differences were observed in the trait parameters between the two species (Table 1). *P. coronopus* reached an observed germination median of 88% at a temperature of 10°C, the experimental temperature closest to the *T*_*o*_G of 11.5°C, while *P. lanceolata* reached an observed germination median of 51% at a temperature of 15°C, the experimental temperature closest to the *T*_*o*_G of 14.4°C (Figure 1A and Supplementary Table S3). Across geographical regions, *P. coronopus* showed similar germination responses to temperature, except the accession from Ci (Supplementary Figure S4f) that showed high germination from 5 to 25°C and from As that showed high germination from 10 to 20°C (Supplementary Figure S4e). *P. lanceolata* also followed similar temperature responses in most regions, except Eg, which showed the lowest germination across temperatures (Supplementary Figure S5d).

**TABLE 1:** Statistical results for the generalized linear model (GLM) tests of the maximum germination (G) and development to the seedling (cotyledon-open) (S) stage performed with the binomial distribution and logit link function with species as a predictor factor using the maximum likelihood estimation method, Wald Chi-Square statistics, and contrast pairwise species.

Species		Traits	*P*-values	Wald Chi-Square
*P. coronopus x P. lanceolata*	Temperature	Germination (%)	**0.007**	1.928
		*T*_*b*_G (°C)	**0.008**	7.109
		θ_*T*_G (°C h)	0.339	0.913
		*T*_*o*_G (°C)	**0.000**	16.47
		Normal seedling development (%)	**0.007**	2,157
		*T*_*b*_S (°C)	**0.001**	11.52
		θ_*T*_S (°C h)	**0.000**	23.997
		*T*_*o*_S (°C)	**0.000**	23.334
	*Osmoticum*	Germination (%)	**0.000**	61.7
		Ψ_*b*_G (MPa)	0.120	2.417
		θ_*H*_G (MPa h)	**0.002**	9.799
		Normal seedling development (%)	**0.000**	387.46
		Ψ_*b*_S (MPa)	**0.037**	4.328
		θ_*H*_S (MPa h)	**0.000**	65.704

*The GLM tests of the base temperatures (*T*_*b*_G and *T*_*b*_S), thermal times (θ_*T*_G and θ_*T*_S), optimum temperatures (*T*_*o*_G and *T*_*o*_S), base water potential (Ψ_*b*_G and Ψ_*b*_S), and hydrotimes (θ_*H*_G and θ_*H*_S) were performed using the above-described method with normal distribution and the identity link function. The significant *p*-values are presented in bold. The overall test results show the Wald Chi-Square test, the degrees of freedom (df) with significant *p*-values in bold, at α = or < 0.05 for each condition.*

Normal seedlings showed significant differences between species (Table 1) with a high response to a broad range of temperatures for *P. coronopus* and a narrower range of temperatures with a high normal seedling response for *P. lanceolata* near the optimum germination temperature (Figure 1B and Supplementary Figures S3, S4 and Supplementary Table S3).

### Osmotic Stress

Final germination for each of the species showed a gradual decrease of germination with decreasing water potentials (Figure 1C). A mild osmotic stress of −0.2 MPa moderately decreased the germination in *P. coronopus* and *P. lanceolata* compared with the control (0.0 MPa) by 25% and 18%, respectively, while a water potential of −0.8 MPa almost fully reduced germination in both species (Figure 1C and Supplementary Table S4). The development of normal seedlings in osmotic solutions reflects the result for germination of each species (Figure 1D and Supplementary Figures S3, S4 and Supplementary Table S4), showing significant differences between species (Table 1) with a strong decrease in normal seedling development at −0.4 MPa in *P. coronopus* when compared with the control. Across geographical regions, the inhibition by osmotic stress on normal seedling development was particularly evident in all *P. lanceolata* accessions (Supplementary Figure S4).

### Germination and Normal Seedling Development Rates

Germination rates and seedling development rates (GR_T_, SR_T_) differed between temperatures and *osmoticum* treatments (GR_W_, SR_W_). Contrary to *P. coronopus* where the GR_T_ and SR_T_ increased steadily from 5 to 30°C (Supplementary Figures S5a,b), *P. lanceolata* showed a transient increase in GR_T_, with the fastest germination and seedling development at 20°C. Moderate osmotic stress only slightly decreased *P. coronopus* GR_W_, but a strong drop was observed at −0.8 MPa. Normal seedling development rates in *osmoticum* (SR_W_) were generally lower than GR_W_, reaching an SR_W_ of zero at −0.8 MPa for both species (Supplementary Figure S5d).

### Base Temperatures, Thermal Times, Base Water Potentials, and Hydrotimes

**FIGURE 2:**
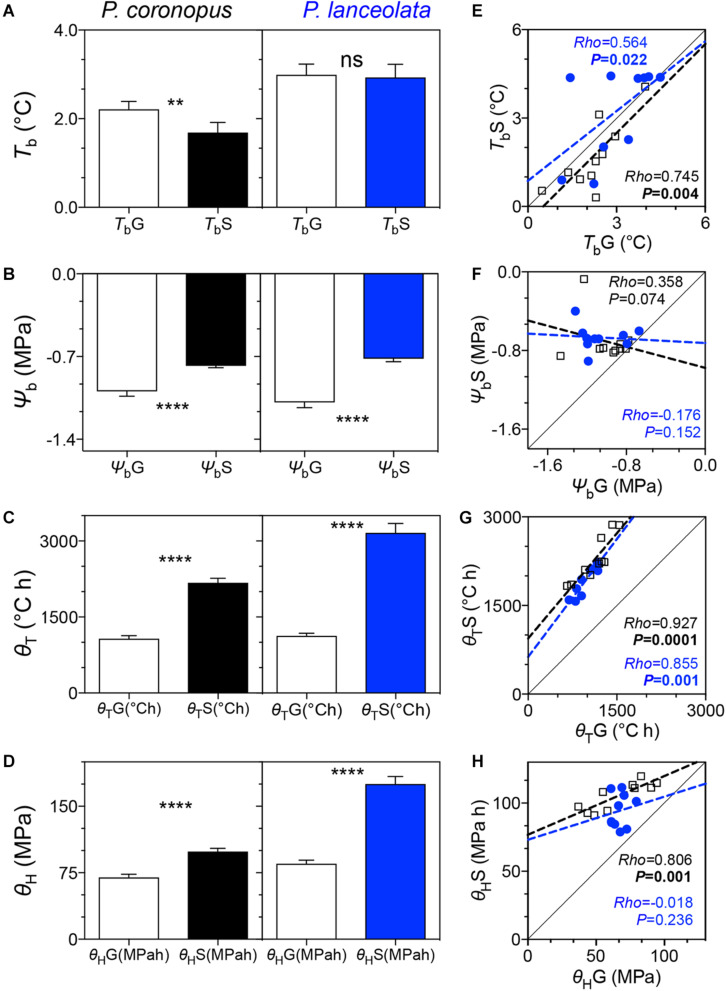
Comparison of base temperature, base water potential, thermal time, hydrotime, and respective linear traits correlations for germination and normal seedling development for 10 accessions of *P. coronopus* (black) and *P. lanceolata* (blue): **(A)** – germination base temperature (*T*_*b*_G) and normal seedling development base temperature (*T*_*b*_S); **(B)** – germination base water potential (Ψ_*b*_G) and normal seedling development base water potential (Ψ_*b*_S); **(C)** – germination thermal time (θ_*T*_G) and normal seedling development thermal time; **(D)** – germination hydrotime (θ_*H*_G) and normal seedling development hydrotime (θ_*H*_S). Linear regressions of the base temperatures for germination (*T*_*b*_G) against the base temperatures for normal seedling development (*T*_*b*_S) **(E)**, base water potential for germination (Ψ_*b*_G) against the base water potential for normal seedling development (Ψ_*b*_S) **(F)**, germination thermal time (θ_*T*_G) against the normal seedling development thermal time (θ_*T*_S) **(G)**, and germination hydrotime (θ_*H*_G) against the normal seedling development hydrotime (θ_*H*_S) **(H)**. Histograms indicate mean ± SEM of two-way ANOVA test comparing the different traits. Asterisks denote the significant difference of *p*-values where: **p = 0.01; ****p = 0.0001; ns, non-significant at α = 0.05. The scatterplot colors represent the mean values of three replicates by species accession.

The germination base temperatures (*T*_*b*_G) showed a small difference for the two species, (*P* = 0.008) (Table 1) with values of 2.0°C for *P. coronopus* and 3.0°C for *P. lanceolata* (Supplementary Table S2). Similarly, base temperatures for normal seedling development (*T*_*b*_S) also differed (*P* = 0.001) (Table 1). *P. coronopus* showed a lower *T*_*b*_S (1.7°C) when compared with the *T*_*b*_G (*P* = 0.005) where *P. lanceolata* showed similar values (Figure 2A and Supplementary Table S2). Despite this, positive correlations were found for both species between the *T*_*b*_G and the *T*_*b*_S (*rho* = 0.564, *P* = 0.022; *rho* = 0.745, *P* = 0.004) (Figure 2E). The base water potential for normal seedling development (Ψ_*b*_S) differed between species (*P* = 0.037), in contrast with the base water potential for germination (Ψ_*b*_G) (Table 1). *P. coronopus* and *P. lanceolata* showed a Ψ_*b*_S that was higher than the Ψ_*b*_G (*P* = 0.0001) (Figure 2B) with values of −0.8 and −1.0 MPa for the former and −0.7 and −1.1 MPa for the latter species (Supplementary Table S2). Unlike the positive correlations for *T*_*b*_G and *T*_*b*_S, both species did not show significant correlations between Ψ_*b*_G and Ψ_*b*_S (Figure 2F). Differences were found in normal seedling development thermal time (θ_*T*_S) between the species but not for germination thermal time (θ_*T*_G), although strong positive correlations were observed between these traits in both species (Figure 2G). Differences were found for both germination hydrotime (θ_*H*_G) and normal seedling development hydrotime (θ_*H*_S), but only *P. coronopus* revealed a significant correlation between both traits (Figure 2H). Both species showed a higher θ_*T*_ and θ_*H*_ for normal seedling development than for germination (Figures 2C,D).

### Factor Analysis of Mixed Data

**FIGURE 3:**
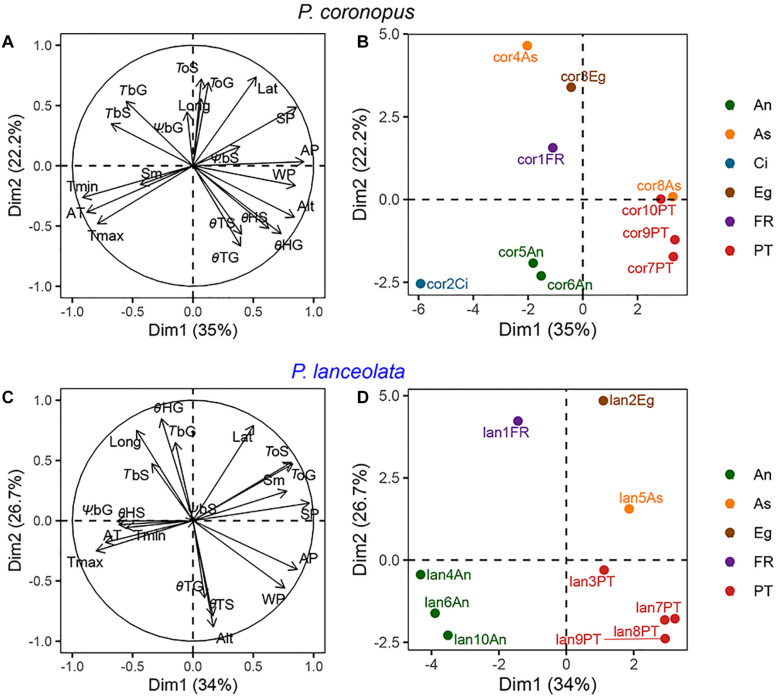
Factor analysis of mixed data (FAMD) analysis with species as categorical variables and physiological traits, provenance climate conditions, and geographical coordinates as continuous variables. **(A,C)** display the quantitative variables that contribute to the total variance; **(B,D)** display accessions plotted by color-coded regions for *P. coronopus* and *P. lanceolata*, respectively. Each accession name contains a species code, accession number, and the region acronym. The environmental temperatures (T_max_, T_min_, and T_a_), environmental precipitation (A_p_, S_p_, and W_p_), geographical coordinates (Alt, Lat, and Long), physiological traits (*T*_*b*_G, *T*_*b*_*S*, *T*_*o*_G, *T*_*o*_S, Ψ_*b*_G, Ψ_*b*_S, θ_*T*_G, θ_*T*_S, θ_*H*_G, and θ_*H*_S), and seed mass (Sm).

An FAMD for *P. coronopus* and *P. lanceolata* was performed using the recorded physiological, geographical, and meteorological traits (Figure 3). The first two dimensions of the FAMD for *P. coronopus* explained 35.0% and 22.2% of the overall variance and for *P. lanceolata* 34.1% and 26.7%.

The geographical and climate conditions showed a strong contribution to the overall variation for both species (Figures 3A,C). The individual factor maps showed similar and clear clustering by geographical region in both species (Figures 3B,D). In the FAMD for *P. coronopus*, the first dimension showed the association of altitude with the base temperature, thermal time, and hydrotime due its high contribution to the variance, but *T*_*b*_S and θ_*H*_G revealed a strong correlation (up to 0.73) with this dimension. In this species, the second dimension explained the effect of the latitude positively correlated with the optimum temperatures, opposite to θ_*T*_G that was negatively correlated (Figure 3A). In the FAMD for *P. lanceolata*, the first dimension showed the optimum temperatures and seed mass similarly affected by climate conditions, particularly summer precipitation. The second dimension showed that thermal times, θ_*T*_G and θ_*T*_S, were similarly affected by altitude and *T*_*b*_G and θ_*H*_G were affected by longitude (Figure 3B).

### Correlations of Physiological, Spatial, and Meteorological Traits

At the collecting sites, the climate conditions vary widely, with only 129 mm annual precipitation in the Canary Islands to 1,432 mm in the north of Portugal and 713 mm in the south of England. The mean annual temperatures range from 20°C in the Canary Islands to around 10°C in the north of Spain and the south of England (Supplementary Table S1).

**FIGURE 4:**
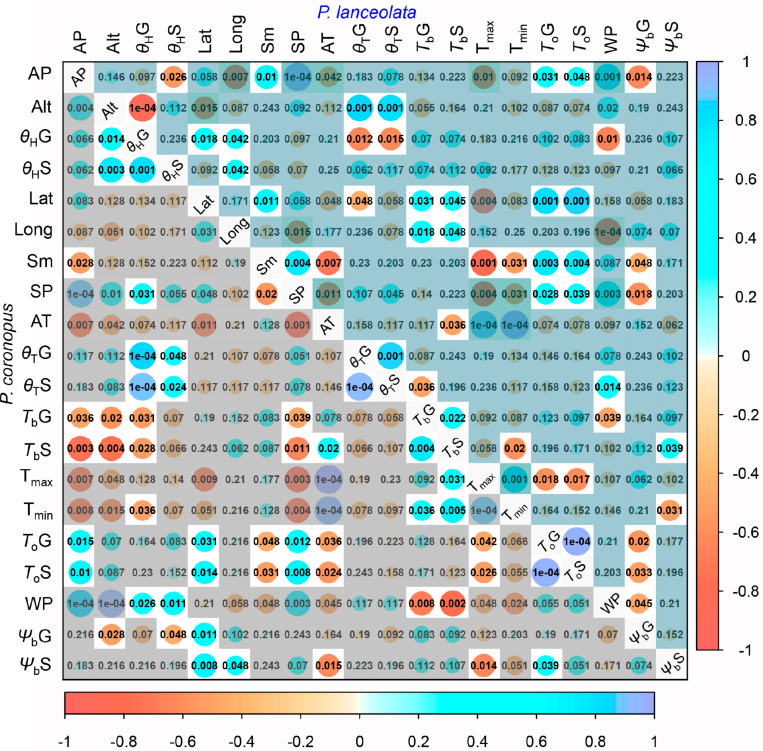
Spearman’s correlation rank diagonal matrix of the geographical, climatic, and experimental data variables (Supplementary Tables S1, S2) for *P. coronopus* (lower) and *P. lanceolata* (upper). Each matrix displays the color-coded correlation coefficients (*rho*) explained by the lateral and bottom color key. The circle sizes represent the *rho* values with the exact *p*-values, shaded if *P* > 0.05 or if not correlated with physiological traits.

**FIGURE 5:**
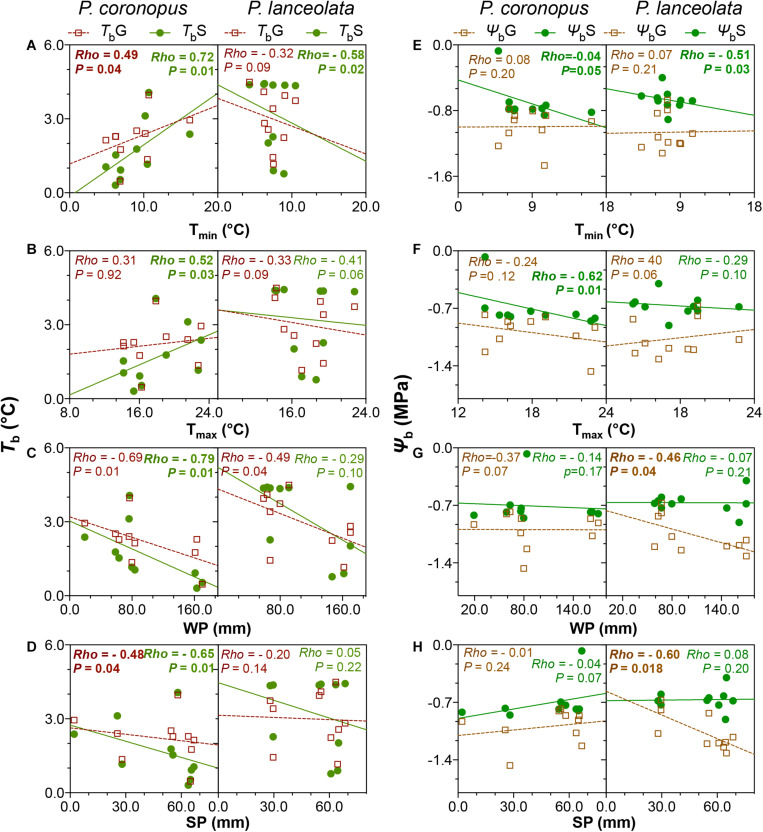
Linear regressions for *P. coronopus* and *P. lanceolata* base temperatures **(A–D)** and base water potentials **(E–H)** against the climate traits: minimum (T_*min*_; **A,E**) and maximum (T_*max*_; **B,F**) temperatures; winter (WP; **C,G**) and summer (SP; **D,H**) precipitation. For each species, the means of three replicate values of 10 accessions were plotted. In each panel, *T*_*b*_G is displayed by brown open squares and brown dotted regression lines, and *T*_*b*_S by green closed symbols and green solid regression lines. *Osmoticum* traits show similar representation. Each panel shows the Spearman’s correlation (*rho*) coefficients and *p*-values.

The bivariate correlation rank coefficients allowed the evaluation of significant correlations between climate and geographical traits with physiological traits for *P. coronopus* and *P. lanceolata* (Figure 4). *P. coronopus* showed significant correlations of altitude with base temperatures and hydrotime for both germination and normal seedling development, although only base water potential for germination shows the same correlation. This contrasts with *P. lanceolata* where only correlations of altitude with the hydrotime for germination and thermal time for germination and normal seedling development were found. In both species, the optimum temperatures (*T*_*o*_G and *T*_*o*_S) were correlated with the latitude, while in *P. coronopus* (but not *P. lanceolata*), the latitude was also correlated with the base water potential (Ψ_*b*_G and Ψ_*b*_S); in *P. lanceolata* (but not *P. coronopus*), latitude showed correlations with base temperatures (*T*_*b*_G and *T*_*b*_S) and with thermal time and hydrotime (θ_*T*_G and θ_*H*_G). *P. coronopus* showed positive correlations of *T*_*b*_G and *T*_*b*_S with environmental temperature traits, while in *P. lanceolata*, only the *T*_*b*_S showed such significant negative correlations. Surprisingly, the T_*min*_ negatively correlated with *P. coronopus* θ_*H*_G (Figure 4). In both species, *T*_*o*_G and *T*_*o*_S correlated with environmental precipitation traits, but only *P. coronopus* showed positive correlations between precipitation and hydrotime traits and negative correlations of all three precipitation traits with the base temperatures. *P. lanceolata* showed negative θ_*H*_G and positive θ_*T*_S correlations with WP. Contrary to *P. coronopus*, the Ψ_*b*_G of *P. lanceolata* was negatively correlated with all precipitation traits (Figures 4, 5G,H). In both species, the Ψ_*b*_S showed correlations with environmental temperature traits: in *P. coronopus* with AT and T_*max*_ and in *P. lanceolata* with T_*min*_ (Figures 4, 5B). The *P. coronopus* seed mass (Sm) was correlated negatively with the environmental precipitation values and with *T*_*o*_G and *T*_*o*_S (Figure 4). Meanwhile, *P. lanceolata* seed mass was positively correlated with SP and AP, as well as with *T*_*o*_G and *T*_*o*_S. A positive correlation for *P. lanceolata* Sm was also found with latitude, and negative correlations were found with the environmental temperature conditions (Figure 4). The correlation analysis demonstrated clear differences in the strategies of these two *Plantago* species to cope with environmental conditions.

### Graph Network Analysis

**FIGURE 6:**
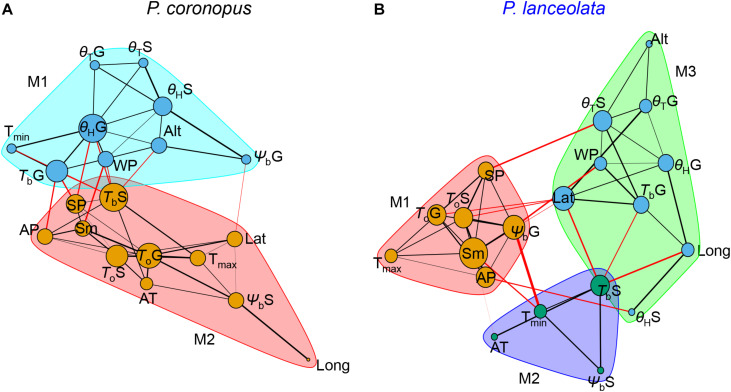
Undirected graph network object of the Spearman’s rank correlation *p*-values of *P. coronopus* **(A)** and *P. lanceolata* **(B)** species. The nodes (circles) correspond to the variable traits, and the edges (lines) correspond to the significant *rho p*-values of two variables in the correlation matrix. The amount of nodes connected depends on the number of significant *p*-values of one variable with all the other variables. The network graph displays edges that are most likely within (black lines) and among (red lines). Node size corresponds to the degree of connections; edge widths correspond to the logarithm of the edge weight of the *p*-value between the two nodes; module communities (M-shaded) are based on edge betweenness (Newman–Girvan algorithm).

*Plantago coronopus* and *P. lanceolata* differed in the established community modules and the connected nodes within and among communities (Figure 6). The network with two *P. coronopus* community modules highlighted that the base temperature for normal seedling development and the germination hydrotime played a key role due to its higher connectivity degree with other traits. The θ_*H*_G trait was more affected by precipitation than temperature conditions, whereas *T*_*b*_S was similarly affected by both climacteric traits (Figure 6A). In contrast, *P. lanceolata* showed that the θ_*H*_G trait connected only with winter precipitation but not summer precipitation (Figure 6B). *P. lanceolata* was organized in three community modules, highlighting a key role for the seed mass due to its higher connectivity degree with other traits within and between communities. This trait was shown to be strongly affected by temperature and precipitation associated with both optimum temperatures for germination and seedling development, as became clear also in the correlation analysis. Contrary to *P. coronopus*, the base temperature for normal seedlings in *P. lanceolata* was associated with both latitude and longitude, and the germination base water potential was mostly associated with the latitude and precipitation values (Figure 6B). These results underline that in these two species, the physiologic traits responded differently to the environmental stimuli.

## Discussion

Our results indicated that the two *Plantago* species exhibited significantly different ecological strategies with clear differences in all analyzed physiologic traits except for thermal time and base water potential for germination (Table 1). *P. coronopus* showed a seedling base temperature (*T*_*b*_S) lower than the germination (*T*_*b*_G), and in *P. lanceolata*, the *T*_*b*_S was not dissimilar from the *T*_*b*_G, implying that normal seedling development does not present an arrested transition between radicle protrusion and normal seedling development. However, a dissimilar thermal time (θ_*T*_) was found for the two species, with lower values for *P. coronopus’* θ_*T*_S and θ_*T*_G (Supplementary Table S2), suggesting that *P. coronopus* can germinate and progress seedling development in lower temperature environments than *P. lanceolata*. Despite a similar range, altitude was correlated negatively with *T*_*b*_G, *T*_*b*_S, and water base (Ψ_*b*_G) in *P. coronopus*, but not in *P. lanceolata*, which suggests functional adaptation of the former species to cold environments. This was supported not only by the positive correlation of *T*_*b*_G and *T*_*b*_S with the environmental T_*min*_ and by the very high seed germination and normal seedling development at 5°C but also the positive correlation in *P. lanceolata* of thermal times with altitude. Results for *P. coronopus* contrast with a recent study testing the initial temperature of seed germination (*T*_*b*_) of 49 species from altitudinal temperate climate, in which Rosbakh and Poschlod (2015) conclude that the *T*_*b*_ is strongly negatively correlated with habitat temperature. Results for *P. lanceolata* were in line, revealing a T_*min*_ negative correlation but only for *T*_*b*_S (Figure 5). Apparently, *P. coronopus* displays the capacity for soil emergence before the winter frost at temperature profiles, which match with first autumn precipitations. Though it should be noted that the current study design focuses on intraspecific as opposed to interspecific variations, as such, the geographical/habitat sampling may have an effect on the outcome. Altitude negatively correlated with the *T*_*b*_ of *P. coronopus*, and the capacity to enable life history transitions at low ambient temperatures could be also explained by the higher precipitation at higher altitude (Figure 4 and Supplementary Table S1). The northern distribution range of *P. coronopus* was restricted to coastal areas, whereas a frequent inland distribution occurs mainly in southern latitudes (Schat, 1981; Kuiper and Bos, 2012), indicating relatively high frost sensitivity. On the other hand, the species showed a continuous increase of germination rate (GR_T_) up to 30°C (Supplementary Figures S3a,b), indicating also high temperature tolerance. This result agrees with reported values for winter and summer weed species where the germination rate increases from 10 to 30°C (Steinmaus et al., 2000). *P. coronopus* demonstrated a *T*_*b*_ that coincides with favorable precipitation events whenever they occur during the “germination” (i.e., sufficiently warm) season, whereas *P. lanceolata* traits seem more suited for germination only in early autumn when the conditions are favorable – avoiding the later season’s low temperatures, which was corroborated by the positive correlations of *T*_*b*_ with the geographical traits and confirmed by the low germination at 5°C. *P. lanceolata* fresh seeds revealed a dormancy degree dependent on the ecotype (van Groenendael, 1986), which explains the observed lower germination values at an optimum temperature and reflects unsuitable environmental conditions, when the probability of seedling survival is low. This is in line with reports stating that seedlings, which have sprouted early in the growth season with a consequently longer time for development, have a lower risk of mortality under subsequent adverse environmental factors (Ross and Harper, 1972; Harper and Harper, 1977).

Contrary to the base temperatures, the base water potential for normal seedling development showed a strong increase, suggesting that the normal seedling development is more limited by water deficit than by the temperature stress. Both species showed an expected higher θ_*H*_S than θ_*H*_G; however, only *P. coronopus* showed a positive correlation between the hydrotime for germination and normal seedling development where the θ_*H*_G seems to be a major trait player. The θ_*H*_ value quantifies the inherent speed of germination, which can vary among species and physiological states being a constant for all seed fractions. Meanwhile, the Ψ_*b*_ threshold values vary among seed lots within a species (Bradford, 2002), indicating that *P. coronopus Ψ*_*b*_S is not the limitation trait in water deficit but the Ψ_*b*_G. This is contrary to *P. lanceolata*, where the seedling conversion was lower in osmotic stress. Indeed, *P. coronopus Ψ*_*b*_S showed a negative correlation with the T_*max*_, indicating higher adaptation of seedlings to osmotic stress in regions with a higher summer temperature, supported by the positive Ψ_*b*_S correlations with the latitude and by the germination and normal seedling percentages under mild stress. This contrasts with *P. lanceolata* that showed a Ψ_*b*_G preference for higher water availability and Ψ_*b*_S for mild temperature environments, as indicated by the negative correlations with these environmental traits. A field study comparing *P. lanceolata* survival in dry and wet site conditions showed that, at dry sites, almost all plants died after a period of drought in the summer of the second year, whereas the wet site had the lowest mortality rate (van Tienderen, 1992). This difference in water stress tolerance for these two species is particularly evident when the conversion from germinated seeds to normal seedlings was observed, with *P. coronopus* close to 40%, whereas *P. lanceolata* showed marginal values at −0.6 MPa (Figure 1D).

It was reported that seed mass is positively associated with germination across a variety of environments (Pearson et al., 2002; Kahmen and Poschlod, 2008). A study in the early eighties in the Netherlands reported a decrease of *P. lanceolata* seed mass from plants grown in dry sites, with the capacity to germinate reduced with decreasing seed sizes, in contrast to wet sites, where plants produced larger seeds that germinated better at lower temperatures and had a lower optimum temperature (van Groenendael, 1986). In agreement, our results revealed that, for this species, seed mass (Sm) increased with summer precipitation (May–October), when the seed development and ripening occurs, and correlated negatively with environmental temperatures, emphasizing that the species preferred lower temperature environments. The observed positive correlation of Sm with the *T*_*o*_ for germination and normal seedling development and the negative correlation with Ψ_*b*_G explain the central position of *P. lanceolata* seed mass in the community module with physiologic traits in the network analysis (Figure 6).

## Conclusion

Our results showed clear species-specific minimum temperature and minimum water requirements for germination and seedling development. Geographical and environmental traits highly contributed to seed functional traits, with the base temperature for normal seedling development as the most central trait in *P. coronopus* and the seed mass associated with similar functional traits in *P. lanceolata.* These results reveal diverse ecological strategies with species-specific importance for functional traits.

## Data Availability Statement

All datasets presented in this study are included in the article/Supplementary Material.

## Author Contributions

AT, PT, and PI conceptualized the work, analyzed the data, discussed the results, and wrote the manuscript. AT conducted the field sampling and laboratory experiments. All authors contributed to the article and approved the submitted version.

## Conflict of Interest

The authors declare that the research was conducted in the absence of any commercial or financial relationships that could be construed as a potential conflict of interest.
