# Supplementary material for: Differential Interspecific Adaptation to Abiotic Stress by Plantago Species
Source: Front Plant Sci. 2020 Nov 5;11:573039. doi: 10.3389/fpls.2020.573039 (PMC7674610; doi:10.3389/fpls.2020.573039)
Supplement: Supplementary file 1 [file Data_Sheet_1.docx]

**Table S1**. Metadata for seeds of five *Plantago* species, collected in six regions at the time of dispersal. Spatial coordinates, (Altitude-meters above sea level [m.a.s.l.], Longitude-decimal degrees - (D.D.) and Latitude-decimal degrees - [D.D.]). WorldClim weather conditions (monthly mean winter precipitation - WP; monthly mean summer precipitation - SP; monthly mean precipitation - AP; Sum annual precipitation - AP_sum_; Minimum temperature - T_min_; Maximum temperature - T_max_; mean annual temperature - AT).

|  |  |  |  |  | Geographical conditions | | |  | WorldClim weather conditions | | | | | | |
| --- | --- | --- | --- | --- | --- | --- | --- | --- | --- | --- | --- | --- | --- | --- | --- |
| Species | Region | Region acronym | Accessions code | Collecting time | Altitude (m.a.s.l.) | Longitude  (D.D.) | Latitude  (D.D.) |  | WP (mm) | SP (mm) | AP (mm) | AP_sum_ (mm) | T_min_ (°C) | T_max_ (°C) | AT (°C) |
| *P. coronopus* | Andalucía (Spain, South) | An | cor5 | June | 403 | -4.88428 | 37.93209 |  | 79.8 | 28.0 | 53.9 | 680 | 10.5 | 22.8 | 16.6 |
| *P. coronopus* | Andalucía (Spain, South) | An | cor6 | June | 613 | -4.35691 | 37.54623 |  | 76.2 | 25.5 | 50.8 | 129 | 10.2 | 21.6 | 15.9 |
| *P. coronopus* | Asturias (Spain, North) | As | cor4 | September | 80 | -4.97879 | 43.45894 |  | 76.8 | 58.2 | 67.5 | 713 | 10.7 | 17.9 | 14.3 |
| *P. coronopus* | Asturias (Spain, North) | As | cor8 | September | 959 | -5.00271 | 43.29151 |  | 83.7 | 66.8 | 75.3 | 810 | 4.9 | 14.2 | 9.5 |
| *P. coronopus* | Canary Islands (Spain) | Ci | cor2 | June | 4 | -13.52947 | 28.97499 |  | 19.3 | 2.2 | 10.8 | 647 | 16.2 | 23.1 | 19.7 |
| *P. coronopus* | England (South) | Eg | cor3 | September | 50 | 0.04678 | 50.78134 |  | 63.3 | 55.5 | 59.4 | 610 | 6.2 | 14.2 | 10.2 |
| *P. coronopus* | France (South) | FR | cor1 | July | 3 | 4.58788 | 43.67373 |  | 58.8 | 54.5 | 56.7 | 1359 | 9.1 | 19.1 | 14.1 |
| *P. coronopus* | Portugal (North) | PT | cor7 | July | 887 | -7.9525 | 41.66728 |  | 161.0 | 65.5 | 113.3 | 903 | 6.9 | 16.0 | 11.5 |
| *P. coronopus* | Portugal (North) | PT | cor9 | July | 1006 | -7.94381 | 41.2741 |  | 170.3 | 65.0 | 117.7 | 1412 | 6.8 | 16.3 | 11.5 |
| *P. coronopus* | Portugal (North) | PT | cor10 | July | 1068 | -7.73216 | 41.48092 |  | 163.2 | 63.8 | 113.5 | 1362 | 6.2 | 15.4 | 10.8 |
| *P. lanceolata* | Andalucía (Spain, South) | An | lan4 | June | 403 | -4.88428 | 37.93209 |  | 79.8 | 28.0 | 53.9 | 680 | 10.5 | 22.8 | 16.6 |
| *P. lanceolata* | Andalucía (Spain, South) | An | lan6 | June | 735 | -3.45198 | 37.78847 |  | 67.2 | 29.7 | 48.4 | 713 | 7.5 | 19.4 | 13.4 |
| *P. lanceolata* | Andalucía (Spain, South) | An | lan10 | June | 1022 | -3.44156 | 37.78512 |  | 67.2 | 29.7 | 48.4 | 1245 | 7.5 | 19.4 | 13.4 |
| *P. lanceolata* | Asturias (Spain, North) | As | lan5 | September | 714 | -6.25345 | 43.11819 |  | 91.3 | 63.5 | 77.4 | 647 | 4.3 | 14.3 | 9.3 |
| *P. lanceolata* | England (South) | Eg | lan2 | September | 50 | 0.04678 | 50.78134 |  | 63.3 | 55.5 | 59.4 | 929 | 6.2 | 14.2 | 10.2 |
| *P. lanceolata* | France (South) | FR | lan1 | July | 3 | 4.58788 | 43.67373 |  | 58.8 | 54.5 | 56.7 | 581 | 9.1 | 19.1 | 14.1 |
| *P. lanceolata* | Portugal (North) | PT | lan3 | July | 389 | -8.24631 | 41.42005 |  | 146.7 | 60.8 | 103.8 | 1357 | 8.9 | 18.6 | 13.8 |
| *P. lanceolata* | Portugal (North) | PT | lan7 | July | 754 | -8.08197 | 41.50206 |  | 161.7 | 64.5 | 113.1 | 1432 | 7.5 | 17.1 | 12.3 |
| *P. lanceolata* | Portugal (North) | PT | lan8 | July | 926 | -7.9543 | 41.59224 |  | 170.2 | 68.5 | 119.3 | 1412 | 6.3 | 15.2 | 10.7 |
| *P. lanceolata* | Portugal (North) | PT | lan9 | July | 1006 | -7.94381 | 41.2741 |  | 170.3 | 65.0 | 117.7 | 581 | 6.8 | 16.3 | 11.5 |

| Species | Region |  |  | Base and optimum temperatures (°C) | | | | Thermal time  (°C h) | | Base water potentials (MPa) | | Hydrotime (MPa h) | |
| --- | --- | --- | --- | --- | --- | --- | --- | --- | --- | --- | --- | --- | --- |
|  |  | Region  acronym | Sm  (mg) | *T*_b_G  (ºC) | *T*_b_S  (ºC) | *T*_o_G  (ºC) | *T*_o_S  (ºC) | *θ*_T_G  (ºCh) | *θ*_T_S  (ºCh) | *Ψ*_b_G  (MPa) | *Ψ*_b_S  (MPa) | *θ*_H_G (MPah) | *θ*_H_S  (MPah) |
| *P. coronopus* | Andalucía (Spain, South) | An | 0.15 | 1.36 | 1.16 | 9.55 | 10.13 | 1289.4 | 2235 | -1.47 | -0.85 | 58.5 | 94.5 |
|  | Andalucía (Spain, South) | An | 0.19 | 2.40 | 3.12 | 9.93 | 9.98 | 656.5 | 1833 | -1.03 | -0.77 | 37.1 | 97.5 |
|  | Asturias (Spain, North) | As | 0.15 | 3.96 | 4.06 | 22.54 | 22.39 | 1543.3 | 2862.7 | -0.86 | -0.73 | 94.7 | 114.8 |
|  | Asturias (Spain, North) | As | 0.13 | 2.14 | 1.05 | 12.38 | 12.23 | 1050.1 | 2018.3 | -1.23 | -0.07 | 77.0 | 113.5 |
|  | Canary Islands | Ci | 0.16 | 2.95 | 2.38 | 7.23 | 7.75 | 692.9 | 1596.3 | -0.93 | -0.82 | 67.4 | 78.8 |
|  | England (South) | Eg | 0.17 | 2.28 | 1.53 | 9.68 | 10.18 | 1067.9 | 2121 | -0.78 | -0.70 | 70.3 | 105.8 |
|  | France (South) | FR | 0.14 | 2.51 | 1.78 | 11.03 | 11.41 | 1182.7 | 2191.3 | -0.81 | -0.78 | 60.9 | 86.5 |
|  | Portugal (North) | PT | 0.16 | 1.76 | 0.92 | 10.83 | 10.56 | 1101.3 | 2153.3 | -0.86 | -0.78 | 91.0 | 114.3 |
|  | Portugal (North) | PT | 0.12 | 0.46 | 0.54 | 10.21 | 10.26 | 1232.7 | 2310.7 | -0.91 | -0.80 | 78.0 | 98.1 |
|  | Portugal (North) | PT | 0.16 | 2.29 | 0.31 | 14.58 | 14.01 | 895.6 | 2433.7 | -1.07 | -0.78 | 61.5 | 86.3 |
|  | * |  | **0.15** | **2.21** | **1.69** | **11.48** | **11.53** | **1071.2** | **2175.5** | **-0.99** | **-0.77** | **69.6** | **99.0** |
| *P. lanceolata* | Andalucía (Spain, South) | An | 1.11 | 3.74 | 4.35 | 12.41 | 12.71 | 1011.5 | 2561.0 | -1.08 | -0.68 | 70.5 | 216.9 |
|  | Andalucía (Spain, South) | An | 1.30 | 1.44 | 4.37 | 10.48 | 11.68 | 1303.3 | 3279.0 | -0.67 | -0.6 | 81.1 | 200.6 |
|  | Andalucía (Spain, South) | An | 1.12 | 3.41 | 2.27 | 11.11 | 12.93 | 800.9 | 3715.0 | -0.79 | -0.73 | 72.0 | 144.6 |
|  | Asturias (Spain, North) | As | 1.45 | 4.49 | 4.39 | 16.09 | 15.94 | 1043.8 | 2595.0 | -1.24 | -0.62 | 96.4 | 149.6 |
|  | England (South) | Eg | 1.61 | 4.1 | 4.4 | 15.94 | 15.99 | 1326.7 | 3440.0 | -0.83 | -0.65 | 133.6 | 155.6 |
|  | France (South) | FR | 1.39 | 3.95 | 4.36 | 16.16 | 16.24 | 1188.3 | 2659.3 | -1.20 | -0.68 | 61.5 | 172.2 |
|  | Portugal (North) | PT | 1.31 | 2.24 | 0.77 | 14.76 | 14.86 | 1000.7 | 3609.3 | -1.19 | -0.73 | 82.2 | 173.4 |
|  | Portugal (North) | PT | 1.77 | 1.16 | 0.9 | 16.29 | 16.06 | 1325.7 | 3788.3 | -1.19 | -0.91 | 82.5 | 109.3 |
|  | Portugal (North) | PT | 1.41 | 2.82 | 4.43 | 15.49 | 15.54 | 1149.9 | 2082.0 | -1.12 | -0.68 | 99.6 | 225.5 |
|  | Portugal (North) | PT | 1.46 | 2.57 | 2.02 | 15.14 | 15.21 | 1126.5 | 3894.0 | -1.32 | -0.4 | 72.1 | 202.8 |
|  | * |  | **1.39** | **2.99** | **2.93** | **14.36** | **14.74** | **1127.7** | **3162.3** | **-1.08** | **-0.71** | **85.2** | **175.0** |

**Table S2.** Metadata and accession-based experimental data for seeds of two *Plantago* species, collected in six regions at the time of dispersal. Seed mass (Sm), base and optimal temperatures (Base temperature of germination - *T*_b_G; Base temperature for normal seedling development - *T*_b_S; Optimum temperature for germination - *T*_o_G; Optimum temperature for seedling development - *T*_o_S) and base water potentials (base water potential for germination - *Ψ*_b_G; base water potential for seedling development - *Ψ*_b_S). Asterisks (*) represents mean by species.

**Table S3.** Medians of germination and normal seedling development in six temperature conditions for each species *per* region. Each region is designated by an acronym: An-Andalucía (Spain, South); As-Asturias (Spain, North); PT-Portugal (North); Eg- England (South); FR- France (South); Ci- Canary Islands (Spain). The asterisk (*) represents the medians at species level.

|  |  |  | Temperature (°C) | | | | | | | | | | | |
| --- | --- | --- | --- | --- | --- | --- | --- | --- | --- | --- | --- | --- | --- | --- |
|  |  |  | 5 | | 10 | | 15 | | 20 | | 25 | | 30 | |
|  | Species | Region | Germination | Normal Seedling Development | Germination | Normal Seedling Development | Germination | Normal Seedling Development | Germination | Normal Seedling Development | Germination | Normal Seedling Development | Germination | Normal Seedling Development |
| Medians (%) | *P. coronopus* | An | 89.9 | 87.0 | 89.0 | 85.0 | 81.0 | 80.0 | 46.0 | 45.0 | 17.0 | 17.0 | 6.0 | 6.0 |
|  |  | PT | 80.0 | 80.0 | 93.6 | 93.6 | 83.7 | 79.6 | 69.4 | 67.3 | 42.0 | 42.0 | 16.7 | 16.7 |
|  |  | As | 18.6 | 18.6 | 57.3 | 56.3 | 60.5 | 59.5 | 60.0 | 58.9 | 51.6 | 49.5 | 41.9 | 35.5 |
|  |  | Eg | 58.3 | 55.6 | 86.5 | 83.8 | 65.9 | 65.9 | 57.1 | 55.9 | 32.4 | 32.4 | 25.0 | 20.8 |
|  |  | FR | 62.0 | 60.0 | 74.0 | 70.0 | 76.0 | 74.0 | 38.0 | 36.0 | 20.0 | 20.0 | 12.0 | 10.0 |
|  |  | Ci | 100.0 | 89.1 | 100.0 | 97.5 | 94.7 | 86.7 | 90.2 | 87.8 | 93.2 | 88.6 | 57.8 | 55.6 |
|  |  | * | 74.5 | 72.3 | 88.2 | 84.7 | 81.5 | 79.6 | 60.0 | 58.7 | 41.2 | 39.0 | 21.5 | 17.6 |
|  | *P. lanceolata* | An | 2.0 | 0.0 | 41.7 | 22.0 | 38.0 | 34.0 | 10.6 | 10.6 | 0.0 | 0.0 | 0.0 | 0.0 |
|  |  | PT | 8.3 | 4.3 | 51.6 | 50.0 | 76.8 | 70.7 | 60.1 | 55.5 | 39.3 | 34.1 | 19.4 | 13.3 |
|  |  | As | 0.0 | 0.0 | 27.1 | 27.1 | 46.9 | 42.9 | 30.4 | 30.4 | 18.8 | 10.2 | 0.0 | 0.0 |
|  |  | Eg | 0.0 | 0.0 | 17.4 | 17.4 | 21.3 | 21.3 | 18.0 | 18.0 | 14.6 | 8.3 | 14.3 | 12.2 |
|  |  | FR | 3.0 | 0.0 | 44.0 | 40.0 | 66.0 | 66.0 | 60.0 | 60.0 | 14.3 | 12.2 | 0.0 | 0.0 |
|  |  | * | 3.1 | 0.0 | 43.0 | 34.7 | 51.0 | 45.9 | 33.7 | 32.6 | 19.4 | 13.6 | 3.1 | 3.1 |

**Table S4.** Medians of germination and normal seedling development in six *osmoticum* conditions for each species *per* region. Each region is designated by an acronym: An-Andalucía (Spain, South); As-Asturias (Spain, North); PT-Portugal (North); Eg- England (South); FR- France (South); Ci- Canary Islands (Spain). The asterisk (*) represents the medians at species level.

|  |  |  | Osmotic potential (MPa) | | | | | | | | | | | |
| --- | --- | --- | --- | --- | --- | --- | --- | --- | --- | --- | --- | --- | --- | --- |
|  |  |  | 0.0 | | -0.2 | | -0.4 | | -0.6 | | -0.8 | | -1.0 | |
|  | Species | Region | Germination | Normal Seedling Development | Germination | Normal Seedling Development | Germination | Normal Seedling Development | Germination | Normal Seedling Development | Germination | Normal Seedling Development | Germination | Normal Seedling Development |
| Medians (%) | *P. coronopus* | An | 81.0 | 80.0 | 78.3 | 77.1 | 48.5 | 42.6 | 14.5 | 10.5 | 4.0 | 0.0 | 0.0 | 0.0 |
|  |  | PT | 83.7 | 79.6 | 67.3 | 63.3 | 38.5 | 27.5 | 10.0 | 6.0 | 0.0 | 0.0 | 0.0 | 0.0 |
|  |  | As | 60.5 | 59.5 | 31.1 | 28.7 | 15.3 | 11.3 | 4.3 | 0.9 | 1.7 | 0.0 | 0.0 | 0.0 |
|  |  | Eg | 65.9 | 65.9 | 21.4 | 21.4 | 7.4 | 3.7 | 4.9 | 0.0 | 0.0 | 0.0 | 0.0 | 0.0 |
|  |  | FR | 84.0 | 84.0 | 50.0 | 50.0 | 17.7 | 15.4 | 5.7 | 1.9 | 0.0 | 0.0 | 0.0 | 0.0 |
|  |  | Ci | 100.0 | 86.7 | 72.7 | 68.2 | 32.7 | 32.5 | 11.9 | 4.1 | 0.0 | 0.0 | 0.0 | 0.0 |
|  |  | * | **81.8** | **79.8** | **56.8** | **56.8** | **27.3** | **21.7** | **8.9** | **3.3** | **0.0** | **0.0** | **0.0** | **0.0** |
|  | *P. lanceolata* | An | 38.0 | 34.0 | 17.0 | 12.5 | 11.1 | 3.9 | 2.2 | 0.0 | 0.0 | 0.0 | 0.0 | 0.0 |
|  |  | PT | 76.8 | 70.7 | 52.9 | 39.4 | 44.8 | 9.4 | 32.3 | 3.1 | 12.4 | 0.0 | 1.0 | 0.0 |
|  |  | As | 46.9 | 42.9 | 25.5 | 23.5 | 27.7 | 6.7 | 10.9 | 0.0 | 3.9 | 0.0 | 0.0 | 0.0 |
|  |  | Eg | 66.0 | 21.3 | 63.3 | 17.3 | 46.0 | 9.3 | 23.4 | 2.0 | 6.7 | 0.0 | 0.0 | 0.0 |
|  |  | FR | 66.0 | 66.0 | 63.3 | 44.0 | 46.0 | 10.6 | 23.4 | 0.0 | 6.7 | 0.0 | 0.0 | 0.0 |
|  |  | * | **51.0** | **45.9** | **33.0** | **24.0** | **29.4** | **8.0** | **12.7** | **0.0** | **4.1** | **0.0** | **0.0** | **0.0** |
