# Supplementary material for: Differential Interspecific Adaptation to Abiotic Stress by Plantago Species
Source: Front Plant Sci. 2020 Nov 5;11:573039. doi: 10.3389/fpls.2020.573039 (PMC7674610; doi:10.3389/fpls.2020.573039)
Supplement: Supplementary file 2 [file Data_Sheet_2.docx]

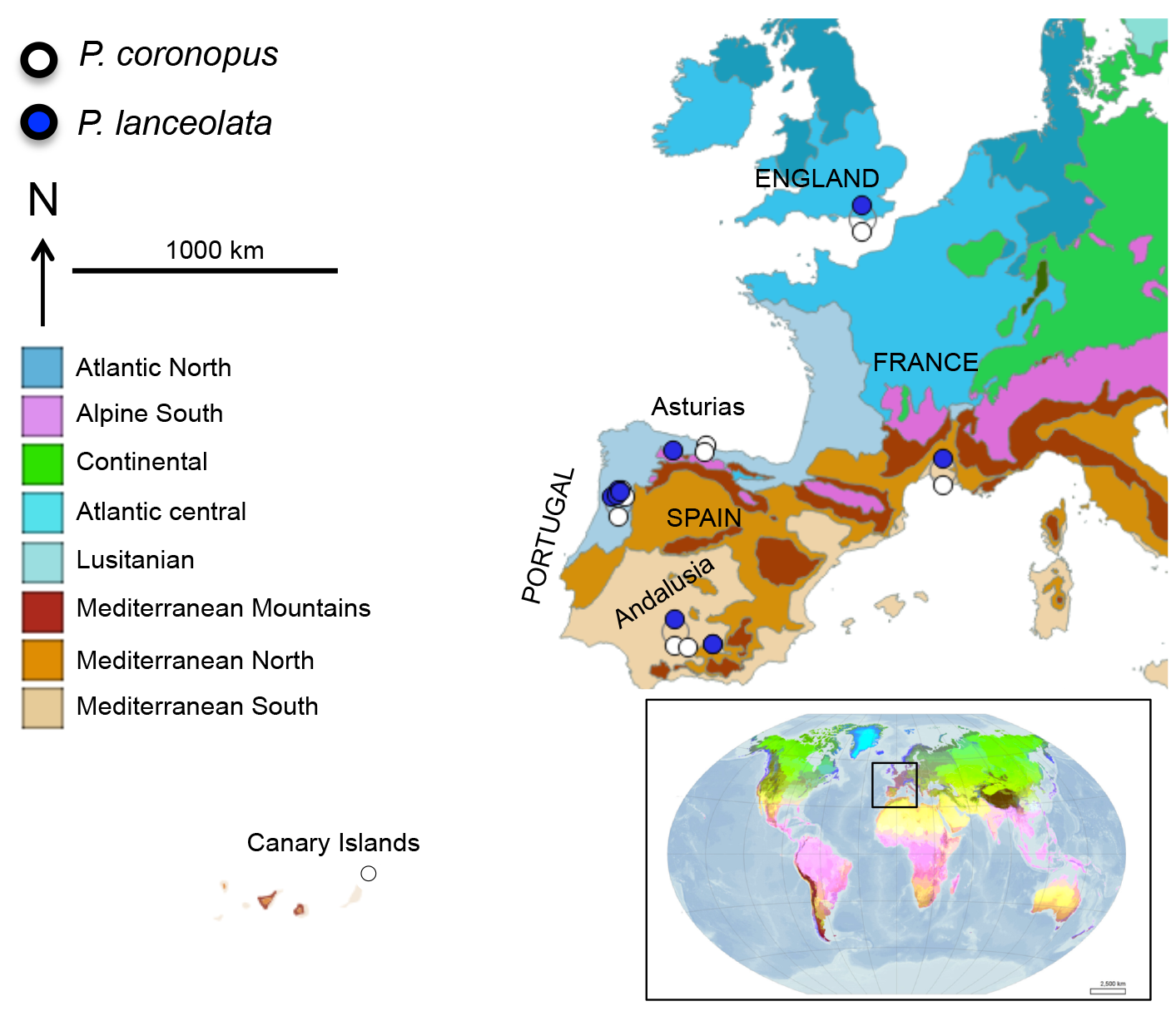


**Figure S1** Map of the collecting regions for the two *Plantago* species (white and blue dots). The map colours represent pedoclimatic conditions of the collecting sites according: The Environmental Stratification of Europe dataset (Metzger, 2018)

**
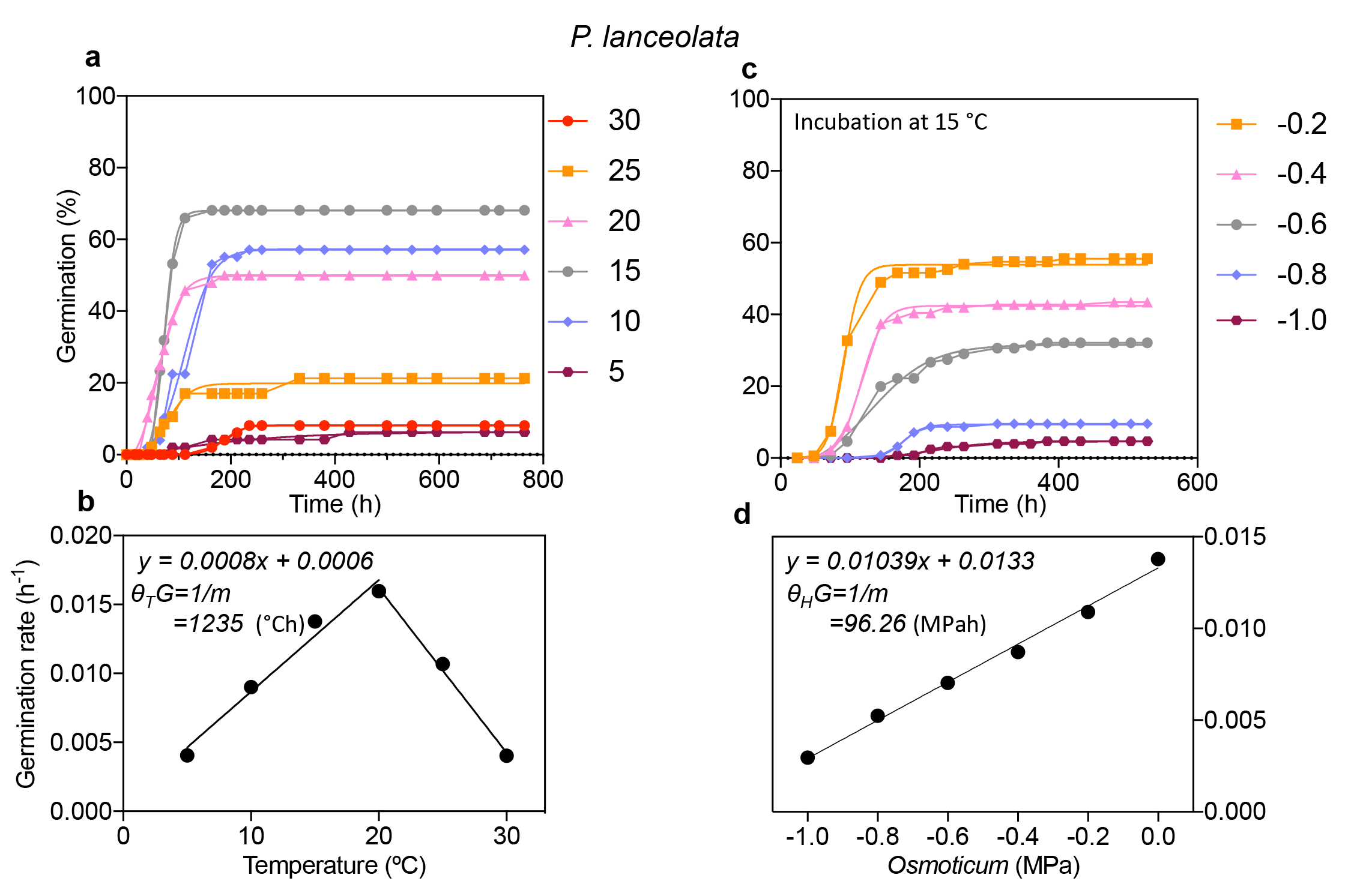
**

**Figure S2**. Example illustration of thermal- and hydro-time calculations from germination responses (% and rate, respectively) of *P. lanceolata* seeds, including response to temperature series 5-30 in 5 ^o^C increments, a and c, respectively. Also, at constant temperature of 15 ^o^C and in response to *osmoticum,* b and d, respectively. Seed germination is modelled via a sigmoidal curve fit of the cumulative germination using the Boltzmann equation, and germination rate (1/*t*_50_ or *GR_50_*) is calculated from the reciprocal of the time to 50% germination. With the range of constant temperatures and *osmoticum*, the reciprocal values that show an increase of *_GR50_* with increasing temperature but below 20 °C were regressed with a linear model to estimate the base temperature (*T*_b_) or the base water potential (*Ψ_b_*) at which the germination rate was equal to zero. The thermal times (*θ*_T_) and hydro times (*θ_H_*) were estimated as the reciprocal of the coefficient of the linear regression, ascertaining the sub-optimal temperature range for *θ*_T_. Trait response in *osmoticum* 0.0 MPa are the same as for 15 °C in water.

**
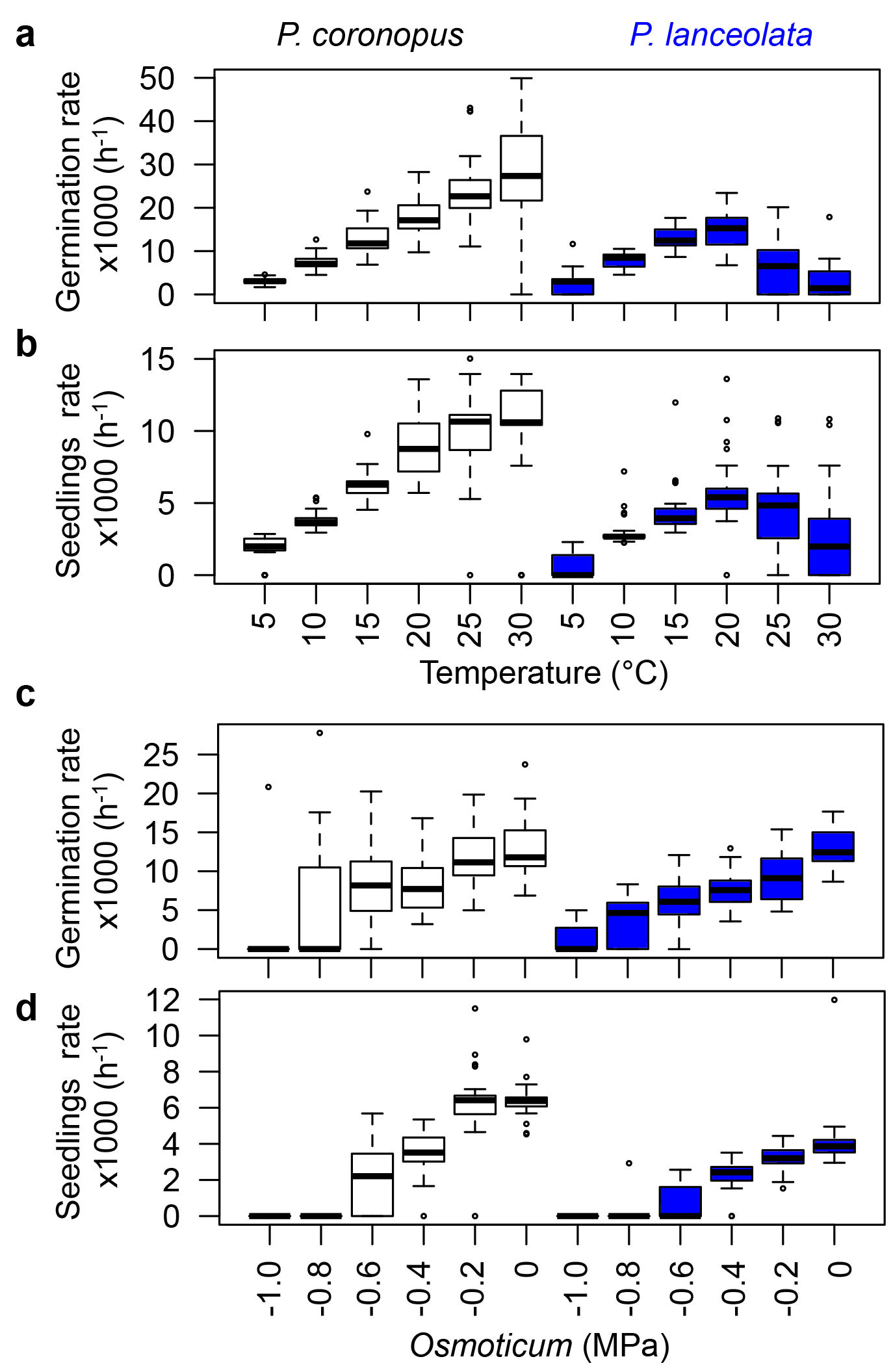
**

**Figure S3** Germination rates and normal seedling development rates (x1000 h^-1^) of *P. coronopus* (white) and *P. lanceolata* (blue), inferred from the reciprocal of the *T*_50_ values. The boxplots show the minimum, first quartile, median, third quartile, maximum and the suspected outliers of the germination rates (panel **a** and **c**) and the seedling development rates (panel **b** and **d**) of six incubation temperatures (**a** and **b**) and *osmoticum* solutions (**c** and **d**)*.* The boxplot colours represent the values of 10 accessions for each species.


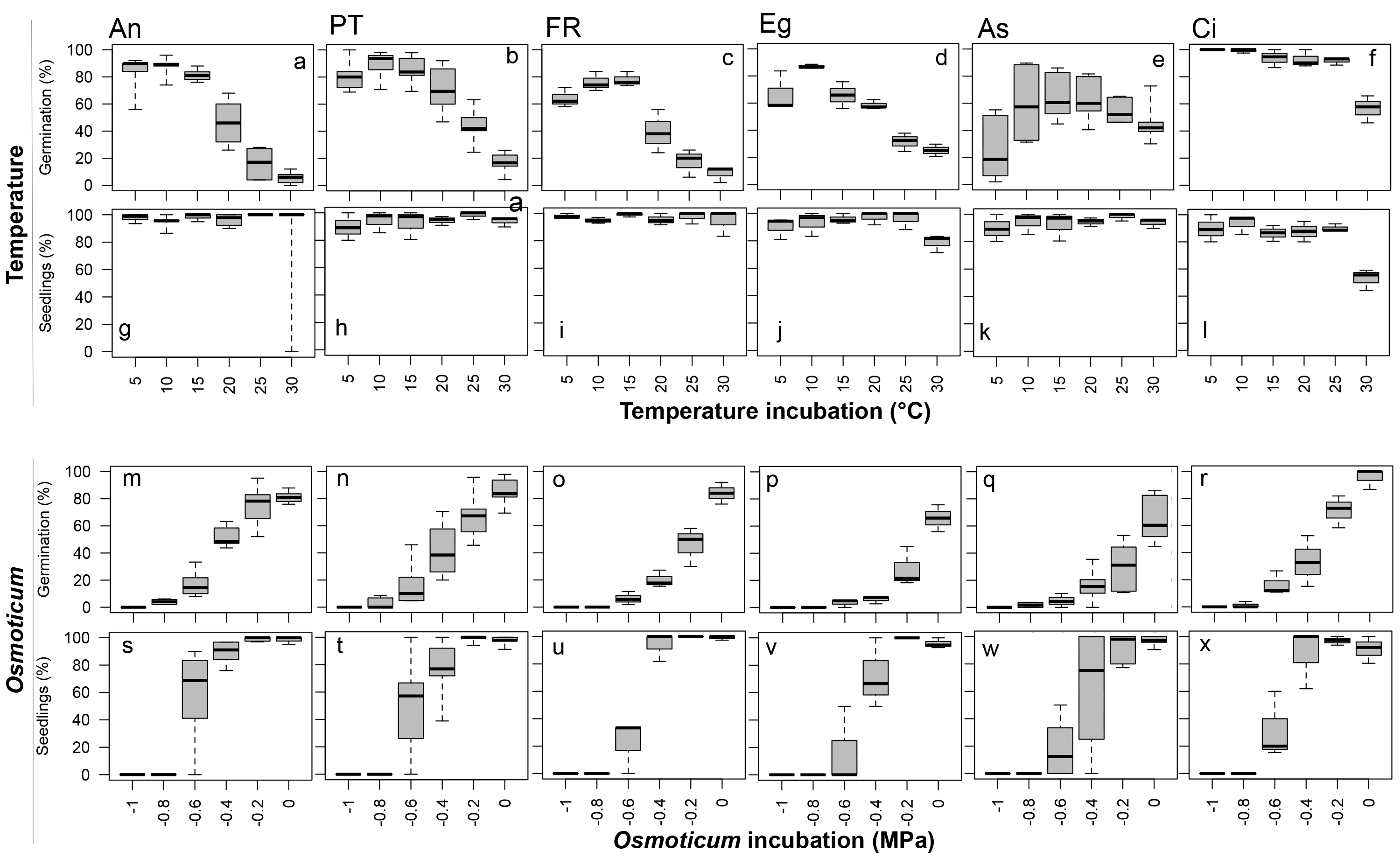


**Figure S4** *P. coronopus* germination percentages and normal seedling percentages per accession from each region incubated at different temperatures and *osmoticum* conditions. The number of species accessions varies between regions, (An = 2; PT = 3; FR=1; Eg = 1; As =2; Ci =1). Each panel shows the minimum, first quartile, median, third quartile, maximum and the suspected outliers. The panels **a**-**f** and **g**-**l** display the germination percentages and normal seedling percentages respectively for the temperatures. The panels **m**-**r** and **s**-**x** display the germination percentages and normal seedling percentages respectively for the *osmoticum* solutions. Each region is represented by an acronym.


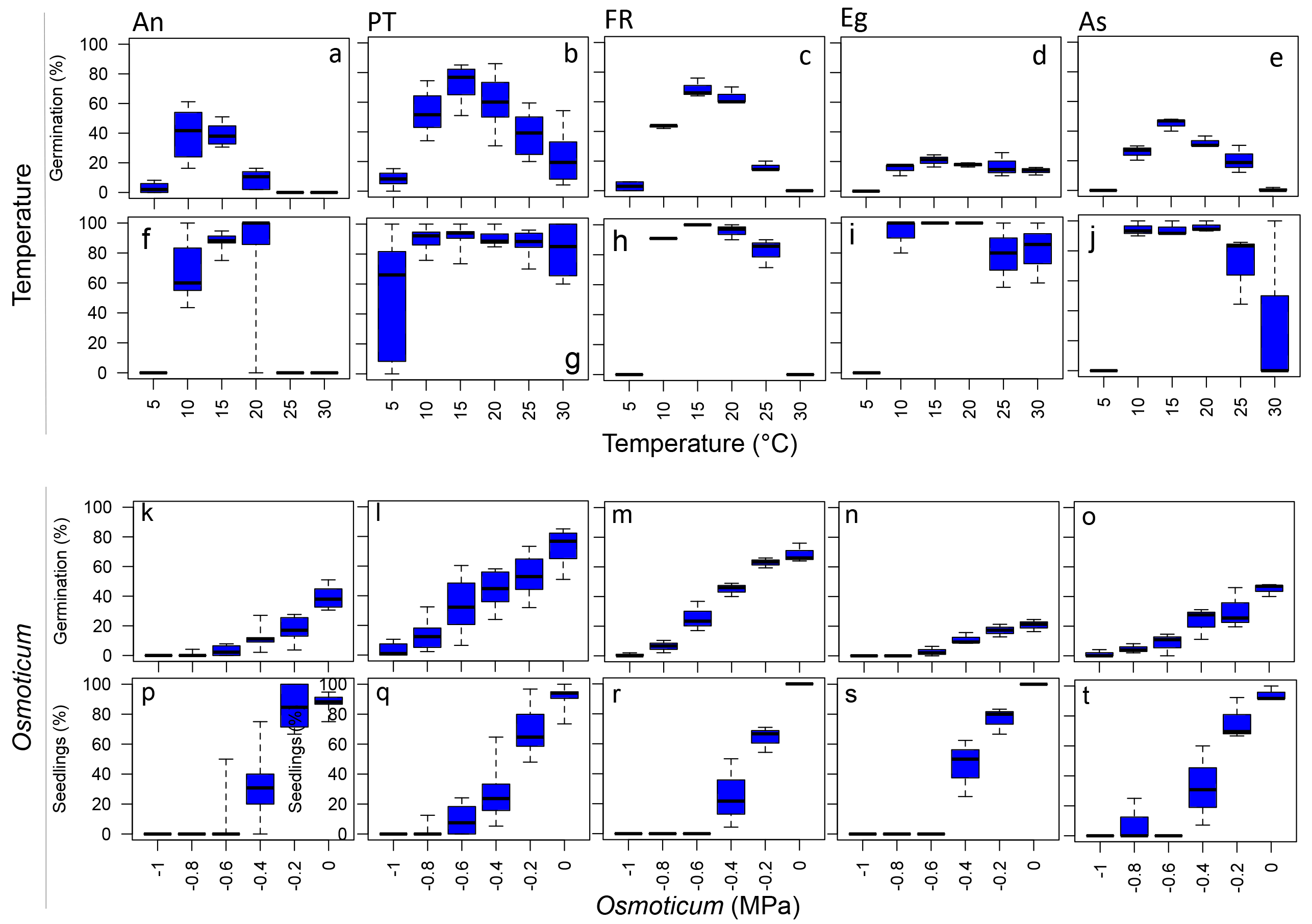


**Figure S5** *P. lanceolata* germination percentages and normal seedling percentages per accession from each represented region incubated at different temperatures and *osmoticum* conditions. The number of species accessions varies between regions (An= 3; PT= 4; FR=1; Eg=1; As=1). Each panel shows the minimum, first quartile, median, third quartile, maximum and the suspected outliers. The panels **a**-**e** and **f**-**j** display the germination percentages and normal seedling percentages respectively for the temperatures. The panels **k**-**o** and **p**-**t** display the germination percentages and normal seedling percentages respectively for the *osmoticum* solutions. Each region is represented by an acronym.
